# Effects of shoulder strapping in patients with stroke: A randomised control trial

**DOI:** 10.4102/sajp.v74i1.430

**Published:** 2018-08-29

**Authors:** Nicolette Comley-White, Witness Mudzi, Eustasius Musenge

**Affiliations:** 1 Department of Physiotherapy, School of Therapeutic Sciences, University of the Witwatersrand, South Africa; 2 School of Public Health, University of the Witwatersrand, South Africa

## Abstract

**Background:**

Disability post stroke remains a global problem, with upper limb involvement playing a key role. Shoulder strapping is one of the techniques used clinically to address this.

**Objectives:**

To compare the effect of two shoulder strapping techniques in patients with stroke.

**Method:**

A longitudinal randomised controlled trial included baseline, weeks one, two and six assessments of 56 participants with upper limb hemiplegia. The participants were assessed for shoulder subluxation, shoulder pain, upper limb motor function and muscle tone. They were randomised into control, longitudinal strapping or circumferential strapping groups.

**Results:**

Longitudinal strapping had a non-significant decrease in shoulder subluxation and pain (*p* > 0.05). Circumferential strapping had no significant effect on any outcomes; however, it prevented the shoulder pain from worsening as much as in the control group (*p* > 0.05). General improvement in upper limb motor function was observed for all three groups.

**Conclusion:**

Trends in improvement showed that longitudinal strapping could be recommended because it positively influenced shoulder subluxation and pain. Even without significant changes, strapping creates awareness of the limb in patients and caregivers and could be of clinical benefit.

**Clinical implication:**

Longitudinal strapping of the shoulder in patients with stroke seems to positively influence shoulder subluxation and pain.

## Introduction

### Background

Stroke continues to be a major health problem world over. Disability post stroke is a common problem affecting functional ability (Brault et al. 2009; Hankey et al. 2007). In South Africa up to 66% of stroke survivors need assistance with at least one activity of daily living (ADL) (Connor et al. 2004). A key factor in post-stroke disability is the involvement of the upper limb. Upper limb problems post-stroke are related to changes in motor function, shoulder subluxation, muscle tone and shoulder pain.

The incidence of shoulder pain post stroke varies from 17% to 87% (Aras et al. 2004; Barlak et al. 2009; Gamble et al. 2002; Lindgren et al. 2007; Ratnasabapathy et al. 2003; Suethanapornkul et al. 2008). Post stroke shoulder pain has been shown to be a contributor to increased hospital length of stay; to negatively affect functional outcomes of patients at discharge from hospital; to restrict ADLs and home ambulation; to reduced quality of life and increased depression (Barlak et al. 2009; Chae et al. 2007; Gamble et al. 2002; Lindgren et al. 2007; Roy et al. 1995).

Up to 77% of patients with stroke have upper limb weakness (Lawrence et al. 2001). The recovery from upper limb hemiplegia is poor, with 62% of patients not gaining any upper limb dexterity by 6 months post stroke (Kwakkel et al. 2003) and 70% of patients with stroke have less than a 50% recovery of the upper limb (Barker, Gill & Brauer 2007). This results in impairment in ADLs and decreased health-related quality of life (Carod-Artal & Egido 2009; Harris & Eng 2007).

Shoulder subluxation post stroke (and subsequent paralysis) arises from compromised shoulder stability. Poor shoulder stability allows gravity to pull the head of the humerus inferiorly, thus stretching the capsule and causing subluxation (Ada & Foongchomcheay 2002). The incidence of shoulder subluxation in patients post stroke ranges from 7% to 81% (Ada & Foongchomcheay 2002) and the evidence is inconclusive in linking shoulder pain to shoulder subluxation (Ada, Foongchomcheay & Canning 2009; Foongchomcheay, Ada & Canning 2006; Kumar & Swinkels 2009; Teasell, Bhogal & Foley 2006; Zorowitz 2001).

Increased muscle tone or spasticity is one of the positive features that arise post stroke as a result of damage to the pyramidal and/or parapyramidal tracts (Ivanhoe & Reistetter 2004; Sheean 2002) and is seen as a common sequela of stroke (Ada, O’Dwyer & O’Neill 2006; Dajpratham et al. 2009; Lundström et al. 2008; Ryu et al. 2010; Sommerfeld et al. 2004; Urban et al. 2010; Watkins et al. 2002; Welmer et al. 2006; Wissel et al. 2010). Spasticity has been linked with lower Barthel Index scores indicating poor functional abilities in ADLs (Urban et al. 2010; Watkins et al. 2002; Wissel et al. 2010). Patients with spasticity have also been shown to have higher incidences of pain, lower scores for quality of life outcome measures, poorer functional gains and more likelihood of institutionalisation (Ryu et al. 2010; Watkins et al. 2002; Wissel et al. 2010).

Despite the high prevalence of post-stroke upper limb complications, there is no clear evidence for definitive treatment techniques that are effective in the management of the shoulder following stroke. Of the various treatment methods that have been proposed in the literature, strapping in particular has stood out as a contentious option. Shoulder strapping is used clinically in patients with stroke, with a variety of techniques being employed; however, the literature is neither conclusively supportive of it, nor definitive in the gold-standard technique (Appel, Mayston & Perry 2011; Appel, Perry & Jones 2014).

The literature describes many different approaches to shoulder strapping (Appel et al. 2011; Chattergee et al. 2016; Griffin & Bernhardt 2006; Hanger et al. 2000; Hayner 2012; Kneeshaw 2002; Morrissey 2000; Pandian et al. 2013; Peters & Lee 2003) with two main trends emerging from the descriptions.

#### Longitudinal strapping method

Variations of this method have been described or used in a range of studies (Chattergee et al. 2016; Hayner 2012; Kneeshaw 2002; Morrissey 2000; Pandian et al. 2013; Peters & Lee 2003). It involves two to three strips of strapping that are applied with a cephalad tension over the anterior, middle and posterior deltoid to end over the shoulder complex, sometimes with an anchor strip applied.

#### Circumferential strapping method

This method has been described and used in two studies (Ancliffe 1992; Griffin & Bernhardt 2006). It involves the application of strapping around the shoulder joint, originating on the clavicle, wrapping around the deltoid to go under the axilla (over a protective pad) and ending on the spine of the scapula.

From these studies, we are shown that strapping the hemiplegic shoulder may have an effect on pain, motor function and shoulder subluxation (Appel et al. 2011; Chattergee et al. 2016; Griffin & Bernhardt 2006; Hanger et al. 2000; Hayner 2012; Pandian et al. 2013; Peters & Lee 2003); however, methodological and sample size limitations have prevented a definitive gold-standard technique from emerging. The aim of this study was therefore to establish the impact of longitudinal and circumferential strapping techniques on a patient’s upper limb muscle tone, shoulder subluxation, shoulder pain and motor function post stroke.

## Method

A longitudinal randomised controlled trial was undertaken. Participants were recruited via consecutive sampling from the medical and neurological wards of two large hospitals in Johannesburg, South Africa, over a 3-year period.

Participants were included in the study if they had a diagnosis of stroke of less than 2 weeks resulting in hemiparesis. They were excluded if they had a previous injury to the shoulder, were medically unstable, had receptive aphasia, decreased consciousness and/or significant visual, perceptual or cognitive problems. The first author regularly screened the relevant wards of the hospitals to check for participants who met the inclusion criteria.

A total of 30 participants had 90% power to detect an effect size of two on the motor assessment scale (effect size for the upper limb components of the motor assessment scale range from 0, 36 to 0, 5 [English et al. 2006]), accounting for a 15% non-compliance and 15% drop-out rate. The significance was set at *p* ≤ 0.05.

A sample size of 30 also had 90% power for all of the other outcome measures.

Participants were randomly assigned to one of three groups: two experimental groups and one control group, using blocked randomisation with the aid of computer-generated random numbers. Allocation to groups was done by a research assistant and the first author was blinded to the group allocation.

### The intervention

Two research assistants applied and removed the strapping as needed. They received training in the strapping techniques by experienced physiotherapists and the techniques were verified in a pilot study. The research assistants were not blinded but the first author, who performed all of the assessments, was. For those in the experimental groups, the research assistants removed the strapping prior to the assessments and reapplied it immediately afterwards.

**FIGURE 1:**
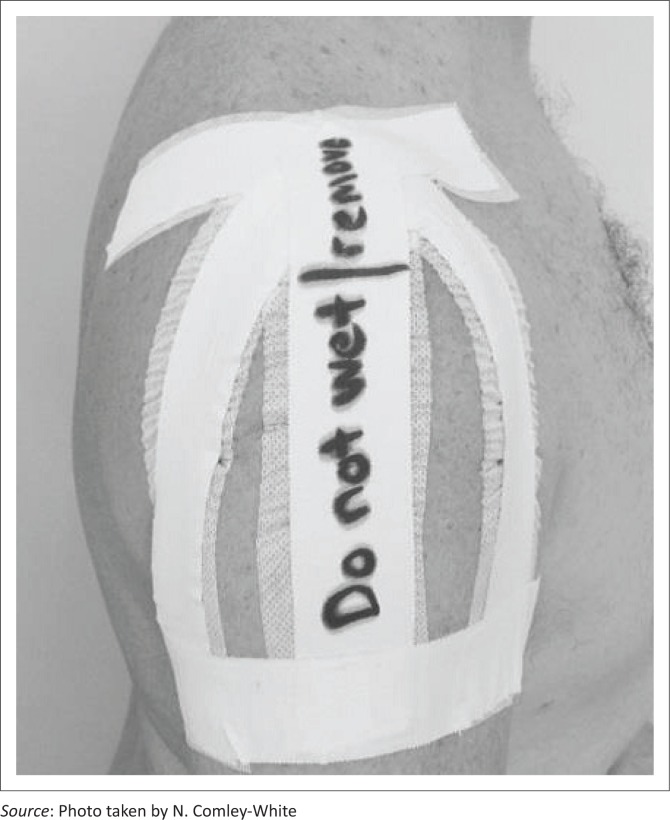
Longitudinal strapping.

The first experimental group received longitudinal shoulder strapping as depicted in Figure 1 (see Appendix 1, Section A1.1. for a detailed description).

**FIGURE 2:**
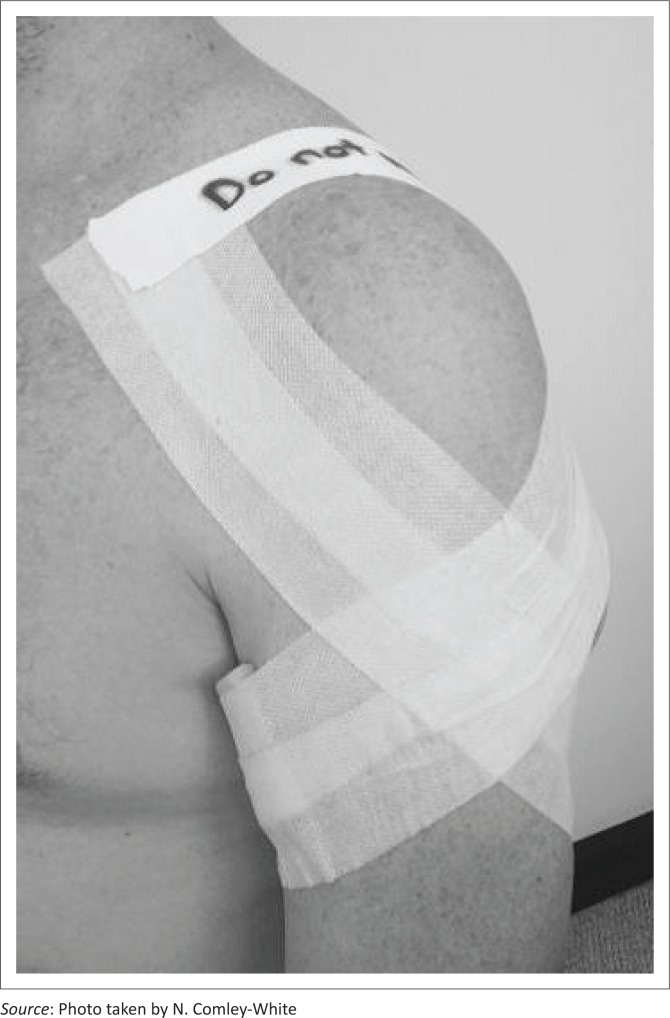
Circumferential strapping.

The second experimental group received circumferential strapping as depicted in Figure 2 (see Appendix 1, Section A1.2. for a detailed description).

The participants in the intervention groups had their shoulders strapped for 2 weeks, with the strapping being changed every 3–4 days. The strapping used was 50-mm-wide Fixomull® Stretch (hypoallergenic) and 38-mm-wide Leukotape P®. The participants in the control group were not strapped.

All the participants (including those in the control group) received standard care from the hospital therapists. This involved education of all patients with hemiplegia on how to perform their own upper limb passive movements (within the limits of pain-free range) using the unaffected arm and the importance of hemiplegic upper limb care, such as handling and positioning of the affected upper limb. In cases where motor function could be elicited, active exercises were given as per standard treatment.

Participants were assessed at baseline, week one, week two and week six. The week six follow up was to assess if any of the potential effects of strapping were retained after the strapping was removed at week two. Changes in upper limb tone were assessed with the modified Ashworth scale, which has fair to very good inter- and intra-rater reliability, with Kappa coefficients ranging from 0.37 to 0.84, depending on the upper limb joint being tested (Ansari et al. 2008; Gregson et al. 1999).

Although x-ray analysis is often seen as the optimal measure of shoulder subluxation (Paci, Nannetti & Rinaldi 2005) factors such as the price, procedure and radiation exposure can frequently make it impractical (Hall, Dudgeon & Guthrie 1995), as was the case in this study. Palpation and measurement of the subacromial space (palpated between the acromion and the superior aspect of the humeral head) using finger breadth has been shown to be a reliable method of clinically measuring shoulder subluxation, with good intra-rater reliability (intra-class correlation coefficient averaging 0.92 across four raters) (Boyd & Torrance 1992). The scale that was used with this method is shown in Table 1 (Hall et al. 1995).

**TABLE 1:** Finger width measurement of shoulder subluxation.

Finger width space of subacromial space	Score
No subluxation	0
½ finger’s width	1
1 finger’s width	2
1½ finger’s width	3
2 finger’s width	4
2½ finger’s width	5

*Source*: Hall, J., Dudgeon, B. & Guthrie, M., 1995, ‘Validity of clinical measures of shoulder subluxation in adults with poststroke hemiplegia’, *The American Journal of Occupational Therapy* 49, 526–533. https://doi.org/10.5014/ajot.49.6.526

Shoulder pain was assessed using the Ritchie articular index: a four-point scale used to describe a patient’s pain in response to passive external rotation of the hemiplegic shoulder (Bohannon & LeFort 1986). The Ritchie articular index is a reliable score (Kappa coefficient of 0.76) (Bohannon & LeFort 1986) and is beneficial to use in patients with communication or cognitive difficulties because it elicits a non-verbal response (Griffin & Bernhardt 2006). Table 2 shows the scoring system used for the Ritchie articular index (Bohannon & LeFort 1986).

**TABLE 2:** Ritchie articular index.

Response	Score
No pain complaint	0
Pain complaint	1
Pain complaint and wincing	2
Pain complaint, wincing and withdrawal. (Withdrawal includes the patient rolling towards their hemiplegic shoulder during testing in order to minimise the force.)	3

*Source*: Bohannon, R. & LeFort, A., 1986, ‘Hemiplegic shoulder pain measured with the Ritchie Articular Index’, *International Journal of Rehabilitation Research* 9, 379–381. https://doi.org/10.1097/00004356-198612000-00009

Motor function was assessed using the upper limb subscales of the motor assessment scale, namely, upper arm function, hand movements and advanced hand movements (Carr et al. 1985). These three components can be used on their own in adult patients with stroke as a valid and reliable tool, with Cronbach’s alpha equal to 0.83 (Lannin 2004).

### Statistical analysis

Demographic data were analysed using descriptive statistics and are presented in tables using frequencies and percentages for the following variables: age, gender and side of stroke. For this study, the tracking of the number of participants presenting with the outcomes that were being measured was important. We therefore used a two-sample test of proportions to determine differences among the groups over the study period. Given the small numbers in the study groups, we used non-parametric tests. Therefore, the overall within-group effect was tested using the Cochran’s *Q* test. Generalised estimating equations were used to determine the overall effects of the intervention over time adjusting for groups as well as using population levels. For all statistical tests, the significance level was set at *p* ≤ 0.05.

### Ethical considerations

Ethical approval was granted by the Human Research Ethics Committee at the University of the Witwatersrand (clearance certificate number: M10903) and informed consent was obtained from all participants prior to the study. Informed consent was given for the photographs.

## Results

**FIGURE 3:**
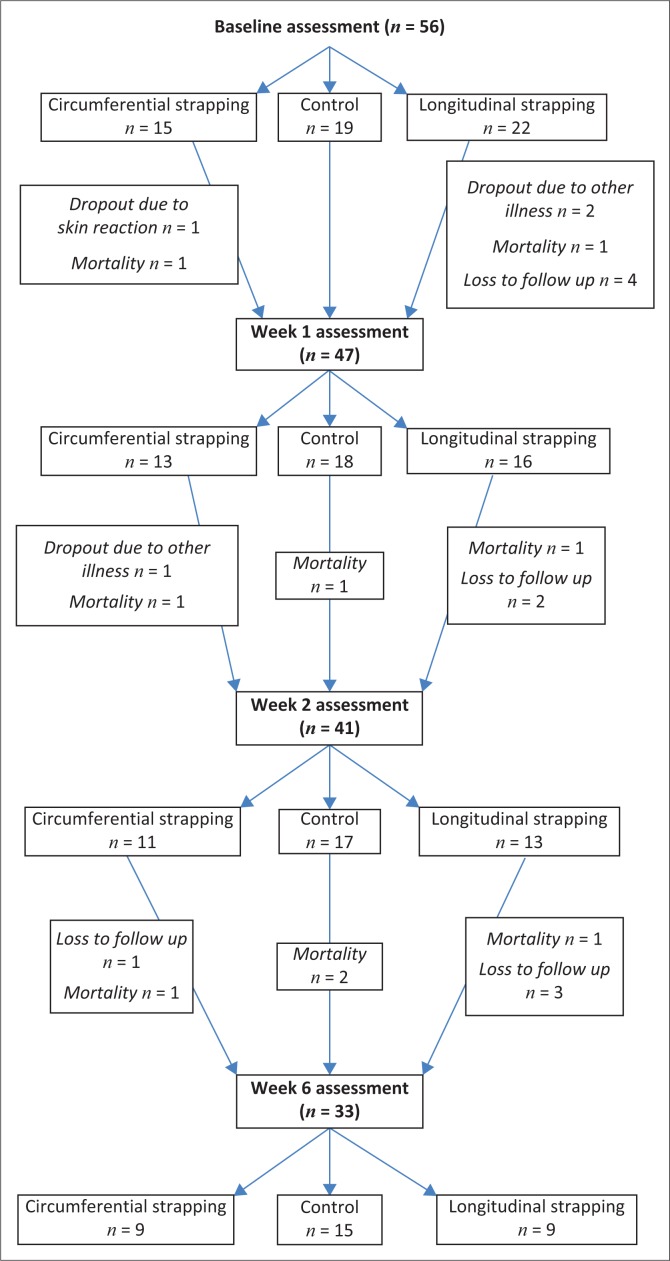
Flowchart showing participant recruitment and assessment.

The demographic details of the participants are shown in Table 3. The majority of the participants were women (51.8%) and the mean age was 49.4 (± 13.8) years. The number of participants at each assessment is shown in Figure 3.

**TABLE 3:** Demographic information (*n* = 56).

Demographic detail	*n* (%)
Male	27 (48.2)
Female	29 (51.8)
Left cerebrovascular accident	22 (39.3)
Right cerebrovascular accident	34 (60.7)
Mean age (SD)	49.4 (±13.8) years

SD, standard deviation; *n*, number of participants. Percentage presented in brackets.

### Outcomes

**FIGURE 4:**
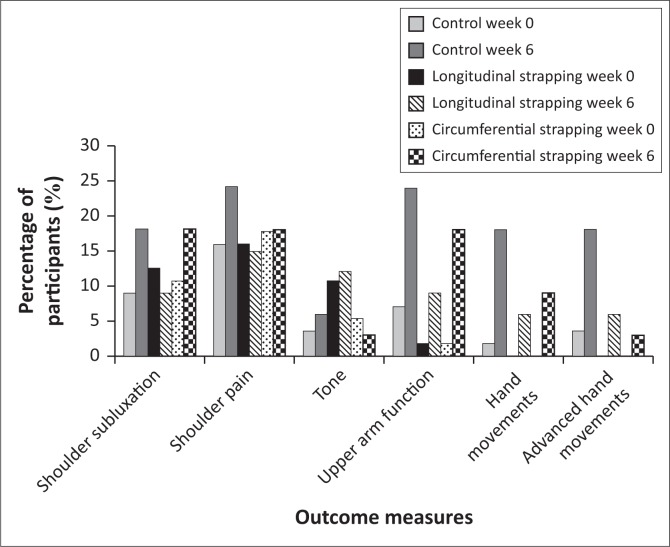
Distribution of participants’ upper limb changes over 6 weeks.

Analysis of the results produced no noteworthy changes between each individual assessment period; thus, the data presented in Figure 4 shows the overall trend in changes across the 6-week period of the study and the following descriptions of the results of the three groups relate to the changes from baseline to week six.

#### Longitudinal strapping versus the control group

Table 4 indicates the changes in outcomes between the longitudinal strapping and the control group over a period of 6 weeks.

**TABLE 4:** Changes in outcomes between the longitudinal strapping and the control group over a 6-week period.

Variables	Score	Longitudinal strapping				Control group			
		Week 0 *n* = 56 *n* (%)	Week 1 *n* = 47 *n* (%)	Week 2 *n* = 41 *n* (%)	Week 6 *n* = 33 *n* (%)	Week 0 *n* = 56 *n* (%)	Week 1 *n* = 47 *n* (%)	Week 2 *n* = 41 *n* (%)	Week 6 *n* = 33 *n* (%)
Shoulder subluxation	0	15 (26.8)	9 (19.1)	8 (19.5)	6 (18.2)	14 (25.0)	10 (21.3)	10 (24.4)	9 (27.3)
	1	3 (5.4)	4 (8.5)	2 (4.9)	1 (3.0)	1 (1.8)	4 (8.5)	1 (2.4)	3 (9.1)
	2	3 (5.4)	3 (6.4)	3 (7.3)	1 (3.0)	3 (5.4)	4 (8.5)	6 (14.6)	3 (9.1)
	3	1(1.8)	0 (0)	0 (0)	1 (3.0)	1(1.8)	0 (0)	0 (0)	0 (0)
	4	0 (0)	0 (0)	0 (0)	0 (0)	0 (0)	0 (0)	0 (0)	0 (0)
	5	0 (0)	0 (0)	0 (0)	0 (0)	0 (0)	0 (0)	0 (0)	0 (0)
Shoulder pain	0	13 (23.2)	10 (21.3)	5 (12.2)	4 (12.1)	10 (17.9)	8 (17.0)	9 (22.0)	7 (21.2)
	1	5 (8.9)	0 (0)	4 (9.8)	0 (0)	8 (14.3)	9 (19.1)	6 (14.6)	3 (9.1)
	2	2 (3.6)	2 (4.3)	4 (9.8)	4 (12.1)	1 (1.8)	1 (2.1)	2 (4.9)	2 (6.1)
	3	2 (3.6)	4 (8.5)	0 (0)	1 (3.0)	0 (0)	0 (0)	0 (0)	3 (9.1)
Tone	0	16 (28.6)	10 (21.3)	8 (19.5)	5 (15.2)	17 (30.4)	14 (29.8)	14 (34.1)	13 (39.4)
	1	2 (3.6)	2 (4.3)	1 (2.4)	0 (0)	1 (1.8)	2 (4.3)	0 (0)	0 (0)
	2	1 (1.8)	1 (2.1)	3 (7.3)	2 (6.1)	1 (1.8)	1 (2.1)	2 (4.9)	1 (3.0)
	3	2 (3.6)	3 (6.4)	1 (2.4)	2 (6.1)	0 (0)	1 (2.1)	1 (2.4)	1 (3.0)
	4	1 (1.8)	0 (0)	0 (0)	0 (0)	0 (0)	0 (0)	0 (0)	0 (0)
	5	0 (0)	0 (0)	0 (0)	0 (0)	0 (0)	0 (0)	0 (0)	0 (0)
Upper arm function	0	21 (37.5)	12 (25.5)	9 (22.0)	6 (18.2)	15 (26.8)	12 (25.5)	10 (24.4)	7 (21.2)
	1	1 (1.8)	3 (6.4)	0 (0)	2 (6.1)	2 (3.6)	0 (0)	0 (0)	1 (3.0)
	2	0 (0)	1 (2.1)	1 (2.4)	0 (0)	0 (0)	1 (2.1)	1 (2.4)	0 (0)
	3	0 (0)	0 (0)	0 (0)	0 (0)	1 (1.8)	2 (4.3)	0 (0)	1 (3.0)
	4	0 (0)	0 (0)	0 (0)	0 (0)	0 (0)	2 (4.3)	1 (2.4)	0 (0)
	5	0 (0)	0 (0)	1 (2.4)	0 (0)	0 (0)	0 (0)	1 (2.4)	0 (0)
	6	0 (0)	0 (0)	2 (4.9)	1 (3.0)	1 (1.8)	1 (2.1)	4 (9.8)	6 (18.2)
Hand movements	0	22 (39.3)	16 (34.0)	10 (24.4)	7 (21.2)	18 (32.1)	15 (31.9)	13 (31.7)	9 (27.3)
	1	0 (0)	0 (0)	0 (0)	0 (0)	0 (0)	1 (2.1)	0 (0)	0 (0)
	2	0 (0)	0 (0)	0 (0)	1 (3.0)	0 (0)	0 (0)	0 (0)	0 (0)
	3	0 (0)	0 (0)	1 (2.4)	0 (0)	1 (1.8)	1 (2.1)	0 (0)	0 (0)
	4	0 (0)	0 (0)	0 (0)	0 (0)	0 (0)	1 (2.1)	2 (4.9)	0 (0)
	5	0 (0)	0 (0)	2 (4.9)	0 (0)	0 (0)	0 (0)	1 (2.4)	2 (6.1)
	6	0 (0)	0 (0)	0 (0)	1 (3.0)	0 (0)	0 (0)	1 (2.4)	4 (12.1)
Advanced hand movements	0	22 (39.3)	16 (34.0)	10 (24.4)	7 (21.2)	17 (30.4)	16 (34.0)	11 (26.8)	9 (27.3)
	1	0 (0)	0 (0)	1 (2.4)	0 (0)	2 (3.6)	0 (0)	3 (7.3)	1 (3.0)
	2	0 (0)	0 (0)	2 (4.9)	1 (3.0)	0 (0)	2 (4.3)	3 (7.3)	3 (9.1)
	3	0 (0)	0 (0)	0 (0)	0 (0)	0 (0)	0 (0)	0 (0)	0 (0)
	4	0 (0)	0 (0)	0 (0)	0 (0)	0 (0)	0 (0)	0 (0)	0 (0)
	5	0 (0)	0 (0)	0 (0)	0 (0)	0 (0)	0 (0)	0 (0)	0 (0)
	6	0 (0)	0 (0)	0 (0)	1 (3.0)	0 (0)	0 (0)	0 (0)	2 (6.1)

Shoulder subluxation: *p* value at week 0 = 0.70; week 1 = 0.97; week 2 = 0.88; week 6 = 0.74

Shoulder pain: *p* value at week 0 = 0.68; week 1 = 0.29; week 2 = 0.43; week 6 = 0.92

Tone: *p* value at week 0 = 0.18; week 1 = 0.33; week 2 = 0.20; week 6 = 0.09

Upper arm function: *p* value at week 0 = 0.11; week 1 = 0.59; week 2 = 0.56; week 6 = 0.34

Hand movements: Final numbers were too small to run statistical tests to calculate *p* value.

Advanced hand movements: Final numbers were too small to run statistical tests to calculate *p* value.

*n*, number of participants. Percentage presented in brackets.

None of the changes in the outcomes reached statistical significance; however, the following findings were of interest.

The percentage of participants who had shoulder subluxation over the study period increased in the control group (from 9% [5/56] to 18% [6/33]) while it declined in the longitudinal strapping group (from 13% [7/56] to 9% [3/33]). Marginally fewer participants in the longitudinal strapping group experienced shoulder pain by the end of the study (5 participants), while there was an increase in the number of participants in the control group who experienced shoulder pain (8 [24%]). Both the control and the longitudinal group experienced an increase in the number of participants who had increased shoulder tone by the end of the study (from 4% [2/56] to 6% [2/33] and from 11% [6/56] to 12% [4/33], respectively). The control group had a greater number of participants with changes in tone than those in the longitudinal group.

Participants in both the control and longitudinal groups exhibited an improvement in upper limb motor function across the study period; however, the control group experienced a greater improvement than the longitudinal group (see Table 4 scores for upper arm function, hand movements, advanced hand movements: a higher percentage of participants scored with a zero at week zero compared to week six).

#### Circumferential strapping versus the control group

Table 5 indicates the changes in outcomes between the circumferential strapping and the control group over a period of 6 weeks.

**TABLE 5:** Changes in outcomes between the circumferential strapping and the control group over a 6-week period.

Variables	Score	Circumferential strapping				Control group			
		Week 0 *n* = 56 *n* (%)	Week 1 *n* = 47 *n* (%)	Week 2 *n* = 41 *n* (%)	Week 6 *n* = 33 *n* (%)	Week 0 *n* = 56 *n* (%)	Week 1 *n* = 47 *n* (%)	Week 2 *n* = 41 *n* (%)	Week 6 *n* = 33 *n* (%)
Shoulder subluxation	0	9 (16.1)	6 (12.8)	5 (12.2)	3 (9.1)	14 (25.0)	10 (21.3)	10 (24.4)	9 (27.3)
	1	2 (3.6)	3 (6.4)	1 (2.4)	4 (12.1)	1 (1.8)	4 (8.5)	1 (2.4)	3 (9.1)
	2	4 (7.1)	3 (6.4)	4 (9.8)	2 (6.1)	3 (5.4)	4 (8.5)	6 (14.6)	3 (9.1)
	3	0 (0)	1 (2.1)	1 (2.4)	0 (0)	1(1.8)	0 (0)	0 (0)	0 (0)
	4	0 (0)	0 (0)	0 (0)	0 (0)	0 (0)	0 (0)	0 (0)	0 (0)
	5	0 (0)	0 (0)	0 (0)	0 (0)	0 (0)	0 (0)	0 (0)	0 (0)
Shoulder pain	0	5 (8.9)	7 (14.9)	4 (9.8)	3 (9.1)	10 (17.9)	8 (17.0)	9 (22.0)	7 (21.2)
	1	4 (7.1)	3 (6.4)	2 (4.9)	1 (3.0)	8 (14.3)	9 (19.1)	6 (14.6)	3 (9.1)
	2	6 (10.7)	3 (6.4)	3 (7.3)	4 (12.1)	1 (1.8)	1 (2.1)	2 (4.9)	2 (6.1)
	3	0 (0)	0 (0)	2 (4.9)	1 (3.0)	0 (0)	0 (0)	0 (0)	3 (9.1)
Tone	0	12 (21.4)	8 (17.0)	7 (17.1)	8 (24.2)	17 (30.4)	14 (29.8)	14 (34.1)	13 (39.4)
	1	1 (1.8)	2 (4.3)	1 (2.4)	0 (0)	1 (1.8)	2 (4.3)	0 (0)	0 (0)
	2	1 (1.8)	2 (4.3)	0 (0)	0 (0)	1 (1.8)	1 (2.1)	2 (4.9)	1 (3.0)
	3	0 (0)	1 (2.1)	3 (7.3)	1 (3.0)	0 (0)	1 (2.1)	1 (2.4)	1 (3.0)
	4	1 (1.8)	0 (0)	0 (0)	0 (0)	0 (0)	0 (0)	0 (0)	0 (0)
	5	0 (0)	0 (0)	0 (0)	0 (0)	0 (0)	0 (0)	0 (0)	0 (0)
Upper arm function	0	14 (25.0)	6 (12.8)	4 (9.8)	3 (9.1)	15 (26.8)	12 (25.5)	10 (24.4)	7 (21.2)
	1	1 (1.8)	5 (10.6)	5 (12.2)	3 (9.1)	2 (3.6)	0 (0)	0 (0)	1 (3.0)
	2	0 (0)	1 (2.1)	0 (0)	0 (0)	0 (0)	1 (2.1)	1 (2.4)	0 (0)
	3	0 (0)	0 (0)	0 (0)	0 (0)	1 (1.8)	2 (4.3)	0 (0)	1 (3.0)
	4	0 (0)	0 (0)	0 (0)	0 (0)	0 (0)	2 (4.3)	1 (2.4)	0 (0)
	5	0 (0)	1 (2.1)	1 (2.4)	2 (6.1)	0 (0)	0 (0)	1 (2.4)	0 (0)
	6	0 (0)	0 (0)	1 (2.4)	1 (3.0)	1 (1.8)	1 (2.1)	4 (9.8)	6 (18.2)
Hand movements	0	15 (26.8)	12 (25.5)	10 (24.4)	6 (18.2)	18 (32.1)	15 (31.9)	13 (31.7)	9 (27.3)
	1	0 (0)	1 (2.1)	1 (2.4)	2 (6.1)	0 (0)	1 (2.1)	0 (0)	0 (0)
	2	0 (0)	0 (0)	0 (0)	0 (0)	0 (0)	0 (0)	0 (0)	0 (0)
	3	0 (0)	0 (0)	0 (0)	0 (0)	1 (1.8)	1 (2.1)	0 (0)	0 (0)
	4	0 (0)	0 (0)	0 (0)	0 (0)	0 (0)	1 (2.1)	2 (4.9)	0 (0)
	5	0 (0)	0 (0)	0 (0)	1 (3.0)	0 (0)	0 (0)	1 (2.4)	2 (6.1)
	6	0 (0)	0 (0)	0 (0)	0 (0)	0 (0)	0 (0)	1 (2.4)	4 (12.1)
Advanced hand movements	0	15 (26.8)	12 (25.5)	11 (26.8)	8 (24.2)	17 (30.4)	16 (34.0)	11 (26.8)	9 (27.3)
	1	0 (0)	1 (2.1)	0 (0)	1 (3.0)	2 (3.6)	0 (0)	3 (7.3)	1 (3.0)
	2	0 (0)	0 (0)	0 (0)	0 (0)	0 (0)	2 (4.3)	3 (7.3)	3 (9.1)
	3	0 (0)	0 (0)	0 (0)	0 (0)	0 (0)	0 (0)	0 (0)	0 (0)
	4	0 (0)	0 (0)	0 (0)	0 (0)	0 (0)	0 (0)	0 (0)	0 (0)
	5	0 (0)	0 (0)	0 (0)	0 (0)	0 (0)	0 (0)	0 (0)	0 (0)
	6	0 (0)	0 (0)	0 (0)	0 (0)	0 (0)	0 (0)	0 (0)	2 (6.1)

Shoulder subluxation: *p* value at week 0 = 0.40; week 1 = 0.61; week 2 = 0.49; week 6 = 0.21

Shoulder pain: *p* value at week 0 = 0.26; week 1 = 0.61; week 2 = 0.39; week 6 = 0.52

Tone: *p* value at week 0 = 0.44; week 1 = 0.33; week 2 = 0.26; week 6 = 0.87

Upper arm function: *p* value at week 0 = 0.24; week 1 = 0.25; week 2 = 0.25; week 6 = 0.52

Hand movements: Final numbers were too small to run statistical tests to calculate *p* value.

Advanced hand movements: Final numbers were too small to run statistical tests to calculate *p* value.

*n*, number of participants. Percentage presented in brackets.

There were no statistically significant changes in the outcomes for the circumferential strapping versus the control participants; however, the following trends were observed. Participants of both the control and circumferential groups had an increase in shoulder subluxation across the study period (from 9% [5/56] to 18% [6/33] and from 11% [6/56] to 18% [6/33], respectively). There was a very slight increase in the number of participants with shoulder pain in the circumferential participants by the end of the study (from 17.9% [10/56] to 18.2% [6/33]), with a more marked increase in the control group (from 16% [9/56] to 24% [8/33]). The distribution of participants in the control group showed an increase in the number of participants with increased shoulder tone over the study period (from 4% [2/56] to 6% [2/33]) while the number of participants with increased tone decreased in the circumferential group (from 5% [3/56] to 3% [1/33]). Motor function across all three subscales improved in both groups by the end of the study period but a larger improvement was shown by the control group, especially in upper arm function (see Table 5 for scores on upper arm function, hand movements and advanced hand movements).

#### Longitudinal strapping versus circumferential strapping

Table 6 indicates the changes in outcomes between the longitudinal strapping and the circumferential strapping group over a period of 6 weeks.

**TABLE 6:** Changes in outcomes between the longitudinal and the circumferential strapping group over a 6 week period.

Variables	Score	Longitudinal strapping				Circumferential strapping			
		Week 0 *n* = 56 *n* (%)	Week 1 *n* = 47 *n* (%)	Week 2 *n* = 41 *n* (%)	Week 6 *n* = 33 *n* (%)	Week 0 *n* = 56 *n* (%)	Week 1 *n* = 47 *n* (%)	Week 2 *n* = 41 *n* (%)	Week 6 *n* = 33 *n* (%)
Shoulder subluxation	0	15 (26.8)	9 (19.1)	8 (19.5)	6 (18.2)	9 (16.1)	6 (12.8)	5 (12.2)	3 (9.1)
	1	3 (5.4)	4 (8.5)	2 (4.9)	1 (3.0)	2 (3.6)	3 (6.4)	1 (2.4)	4 (12.1)
	2	3 (5.4)	3 (6.4)	3 (7.3)	1 (3.0)	4 (7.1)	3 (6.4)	4 (9.8)	2 (6.1)
	3	1 (1.8)	0 (0)	0 (0)	1 (3.0)	0 (0)	1 (2.1)	1 (2.4)	0 (0)
	4	0 (0)	0 (0)	0 (0)	0 (0)	0 (0)	0 (0)	0 (0)	0 (0)
	5	0 (0)	0 (0)	0 (0)	0 (0)	0 (0)	0 (0)	0 (0)	0 (0)
Shoulder pain	0	13 (23.2)	10 (21.3)	5 (12.2)	4 (12.1)	5 (8.9)	7 (14.9)	4 (9.8)	3 (9.1)
	1	5 (8.9)	0 (0)	4 (9.8)	0 (0)	4 (7.1)	3 (6.4)	2 (4.9)	1 (3.0)
	2	2 (3.6)	2 (4.3)	4 (9.8)	4 (12.1)	6 (10.7)	3 (6.4)	3 (7.3)	4 (12.1)
	3	2 (3.6)	4 (8.5)	0 (0)	1 (3.0)	0 (0)	0 (0)	2 (4.9)	1 (3.0)
Tone	0	16 (28.6)	10 (21.3)	8 (19.5)	5 (15.2)	12 (21.4)	8 (17.0)	7 (17.1)	8 (24.2)
	1	2 (3.6)	2 (4.3)	1 (2.4)	0 (0)	1 (1.8)	2 (4.3)	1 (2.4)	0 (0)
	2	1 (1.8)	1 (2.1)	3 (7.3)	2 (6.1)	1 (1.8)	2 (4.3)	0 (0)	0 (0)
	3	2 (3.6)	3 (6.4)	1 (2.4)	2 (6.1)	0 (0)	1 (2.1)	3 (7.3)	1 (3.0)
	4	1 (1.8)	0 (0)	0 (0)	0 (0)	1 (1.8)	0 (0)	0 (0)	0 (0)
	5	0 (0)	0 (0)	0 (0)	0 (0)	0 (0)	0 (0)	0 (0)	0 (0)
Upper arm function	0	21 (37.5)	12 (25.5)	9 (22.0)	6 (18.2)	14 (25.0)	6 (12.8)	4 (9.8)	3 (9.1)
	1	1 (1.8)	3 (6.4)	0 (0)	2 (6.1)	1 (1.8)	5 (10.6)	5 (12.2)	3 (9.1)
	2	0 (0)	1 (2.1)	1 (2.4)	0 (0)	0 (0)	1 (2.1)	0 (0)	0 (0)
	3	0 (0)	0 (0)	0 (0)	0 (0)	0 (0)	0 (0)	0 (0)	0 (0)
	4	0 (0)	0 (0)	0 (0)	0 (0)	0 (0)	0 (0)	0 (0)	0 (0)
	5	0 (0)	0 (0)	1 (2.4)	0 (0)	0 (0)	1 (2.1)	1 (2.4)	2 (6.1)
	6	0 (0)	0 (0)	2 (4.9)	1 (3.0)	0 (0)	0 (0)	1 (2.4)	1 (3.0)
Hand movements	0	22 (39.3)	16 (34.0)	10 (24.4)	7 (21.2)	15 (26.8)	12 (25.5)	10 (24.4)	6 (18.2)
	1	0 (0)	0 (0)	0 (0)	0 (0)	0 (0)	1 (2.1)	1 (2.4)	2 (6.1)
	2	0 (0)	0 (0)	0 (0)	1 (3.0)	0 (0)	0 (0)	0 (0)	0 (0)
	3	0 (0)	0 (0)	1 (2.4)	0 (0)	0 (0)	0 (0)	0 (0)	0 (0)
	4	0 (0)	0 (0)	0 (0)	0 (0)	0 (0)	0 (0)	0 (0)	0 (0)
	5	0 (0)	0 (0)	2 (4.9)	0 (0)	0 (0)	0 (0)	0 (0)	1 (3.0)
	6	0 (0)	0 (0)	2 (4.9)	1 (3.0)	0 (0)	0 (0)	0 (0)	0 (0)
Advanced hand movements	0	22 (39.3)	16 (34.0)	10 (24.4)	7 (21.2)	15 (26.8)	12 (25.5)	11 (26.8)	8 (24.2)
	1	0 (0)	0 (0)	1 (2.4)	0 (0.0)	0 (0)	1 (2.1)	0 (0)	1 (3.0)
	2	0 (0)	0 (0)	2 (4.9)	1 (3.0)	0 (0)	0 (0)	0 (0)	0 (0)
	3	0 (0)	0 (0)	0 (0)	0 (0)	0 (0)	0 (0)	0 (0)	0 (0)
	4	0 (0)	0 (0)	0 (0)	0 (0)	0 (0)	0 (0)	0 (0)	0 (0)
	5	0 (0)	0 (0)	0 (0)	0 (0)	0 (0)	0 (0)	0 (0)	0 (0)
	6	0 (0)	0 (0)	0 (0)	1 (3.0)	0 (0)	0 (0)	0 (0)	0 (0)

Shoulder subluxation: *p* value at week 0 = 0.61; week 1 = 0.97; week 2 = 0.43; week 6 = 0.16.

Shoulder pain: *p* value at week 0 = 0.12; week 1 = 0.64; week 2 = 0.92; week 6 = 0.63.

Tone: *p* value at week 0 = 0.61; week 1 = 0.96; week 2 = 0.92; week 6 = 0.11.

Upper arm function: (*p* value at week 0 = 0.78; week 1 = 0.11; week 2 = 0.11; week 6 = 0.16.

Hand movements: Final numbers were too small to run statistical tests to calculate *p* value.

Advanced hand movements: Final numbers were too small to run statistical tests to calculate *p* value.

*n*, number of participants. Percentage presented in brackets.

While none of the changes held any statistical significance, the following was noted.

During the study period, the distribution of participants with shoulder subluxation increased in the circumferential strapping group (from 11% [6/56] to 18% [6/33]) and decreased in the longitudinal group (from 13% [7/56] to 9% [3/33]). The distribution of participants with shoulder pain marginally decreased (from 16% [9/56] to 15% [5/33]) and increased (from 17.9% [10/56] to 18.2% [6/33]) for the longitudinal and circumferential groups, respectively. The distribution of participants with increased shoulder tone rose in the longitudinal group, over the study period (from 11% [6/56] to 12% [4/33]), while the circumferential participants decreased (from 5% [3/56] to 3% [1/33]). The scores showed that participants of both intervention groups showed improvement across all three subscales over the study period with the circumferential group showing greater improvement for upper arm function and hand movements (see Table 6 for scores on upper arm function, hand movements and advanced hand movements). Table 7 shows a summary of the trend that was observed in each group over time.

**TABLE 7:** Observed trends across the study period for increases (↑) or decreases (↓) in outcome measures for all groups.

Variables	Shoulder subluxation	Shoulder pain	Tone	Motor function
Control	↑	↑	↑	↑
Longitudinal	↓	↓ (Marginal)	↑	↑
Circumferential	↑	↑ (Marginal)	↓	↑

## Discussion

This study aimed to compare the effect of two shoulder strapping techniques, longitudinal and circumferential, in patients with stroke. The most important finding was that longitudinal strapping positively influenced the participants’ shoulder subluxation and pain post stroke. It should be noted that in our study, not only did the longitudinal group have slightly less shoulder pain at the end of the study but the number of participants with pain in the control group had increased, leading one to consider the effects of the strapping in preventing the worsening of shoulder pain post stroke. The longitudinal strapping also resulted in a decrease in the percentage of participants with shoulder subluxation. The circumferential technique was ineffective in preventing shoulder subluxation post stroke. This may be because the technique possibly creates very little anti-gravity tension and hence clinically one would not expect to see the circumferential strapping having a positive effect on shoulder subluxation. However, if one considers the cephalad tension applied in the longitudinal method, it makes clinical sense that the longitudinally strapped participants were less likely to develop shoulder subluxation.

One could attribute the effect of strapping on shoulder pain to the increased awareness of the affected limb, leading to more careful handling by the caregivers. It could also be considered that the strapping provided cutaneous stimulation through the large fibres (C fibres), which would be introducing a competing sensation to pain and hence the perception that pain has decreased (Melzack & Wall 1965). Furthermore, one could consider the decrease in shoulder subluxation in the longitudinally strapped participants to be a contributing factor to the better pain outcomes in the intervention group.

Longitudinal strapping played no role in preventing increased upper limb muscle tone post stroke. One would not necessarily expect to see strapping inhibit muscle tone changes post stroke as there is no clinical explanation behind it. The only role perhaps that strapping could be attributed to is in decreasing pain, which in turn could influence tone positively. This may have been the case for the participants with circumferential strapping as they showed a slight decrease in tone while not experiencing increased shoulder pain. However, the changes between the two groups did not have statistical significance and thus one cannot categorically state that circumferential strapping prevented an increase in upper limb tone post stroke.

With time, motor function begins to improve in the upper limb post stroke because of natural recovery (Newman 1972). This was encountered when all groups showed an improvement in motor function across the study period. There was no statistical evidence in this study to show that strapping had an effect of motor function, which was a similar finding in randomised control trials using the circumferential technique (Griffin & Bernhardt 2006) and longitudinal technique (Pandian et al. 2013).

The circumferential strapping technique was used in two other studies, both of which showed that there was a delay in the onset of pain in the strapped participants (Ancliffe 1992; Griffin & Bernhardt 2006). Similarly, our study found that those participants strapped circumferentially had less of an increase in pain compared to those in the control group.

As with the longitudinal strapping in this study, two other studies using a similar technique found a trend in improvement in shoulder subluxation; however, it should be noted that the one study had only eight participants upon completion (Hayner 2012) and that in the other study the changes in subluxation were not statistically significant (Chatterjee et al. 2016). No other literature, to the authors’ knowledge, indicates the effects of longitudinal strapping on shoulder subluxation.

The largest post-stroke shoulder strapping, randomised control trial to date compared longitudinal shoulder strapping with sham strapping in patients less than 48 h post stroke (Pandian et al. 2013). For this study, 162 participants were strapped and assessed for 2 weeks and a follow-up at 1 month. The authors found that although there was a trend towards decreased pain in the intervention group, the difference compared to the control group was not statistically significant (Pandian et al. 2013). Chatterjee et al. (2016) applied a similar technique of shoulder strapping to patients with stroke and found a statistically significant improvement in shoulder pain. These findings were similar to those of our study’s trend in longitudinal strapping decreasing shoulder pain.

When comparing the results of each intervention group against each other, there was no statistically significant difference between the two groups. Although not part of our study, it was noted that the research assistants found the longitudinal technique easier to apply and that it was less time intensive than the circumferential technique. Additionally, the circumferential technique required padding material (over and above the strapping) and this was a further resource and cost. These are inconsequential considerations if the circumferential technique had been found to be far superior in its results compared to the longitudinal technique. However, it was not and thus the longitudinal technique, with its positive though non-significant effect on shoulder subluxation and pain, would appear to be the preferred method of the two. This is backed by the positive outcomes of other studies using variations of the longitudinal method (Chattergee et al. 2016; Hayner 2012; Pandian et al. 2013).

Upper limb problems post stroke continue to be a major health issue. Although shoulder strapping is largely used post stroke, its efficacy still remains uncertain. The potential value of longitudinal shoulder strapping in combating shoulder subluxation and pain should be explored in further studies.

## Limitations and challenges

Once data collection began, it became apparent to the first author that finding participants who met the inclusion criteria was a challenge. The participants were required to have hemiplegia but were excluded for receptive aphasia or any significant visual, perceptual or cognitive problems. Combining these inclusion and exclusion criteria considerably diminished the availability of participants. Similarly, Appel et al. (2011) found that targeting such a specific population resulted in only recruiting 10% of stroke admissions for their study on shoulder strapping.

Despite increasing the catchment area for participants by expanding to surrounding hospitals, it took over 3 years to include 56 participants. Of the 56 participants, 33 reached the final assessment, meeting the sample size calculated. The two main reasons for loss of participants were morbidity (16% of 56 participants) and loss to follow-up (18% of 56 participants). The high morbidity rate was similar to previous results found in a Johannesburg hospital in patients with stroke, whereby 26% of patients with stroke died within 3 months post discharge (Mudzi, Stewart & Musenge 2012).

The loss to follow up was mainly because of participant transport problems as the majority of the participants used public transport which was costly and difficult to access within the province, especially post stroke. Of those lost to follow up, many participants left Johannesburg to join family who would be able to care for them. The first author had funding to help with the cost of transport and she often travelled to access participants; however, some remained inaccessible and thus were unable to complete the full study period.

Although the sample size was sufficiently powered, the final number of participants still leaves an overall small sample size, making generalisation to the entire stroke population difficult.

## Conclusion

Overall, the study showed trends in changes in the shoulder post stroke but no significant differences were found among the groups in any of the outcomes. When weighing one type of strapping up against another, the longitudinal technique positively influenced shoulder subluxation and shoulder pain (marginally).

Although the study produced overall results that did not have statistical significance, one cannot discredit the use of strapping. Even if strapping had a purely placebo effect, it possibly may still serve a purpose by creating awareness in the patient, caregivers and medical personal and thus ensure more cautious handling of the affected upper limb. As the rehabilitation of the upper limb is a challenging area, any technique that may aid recovery should be considered.

## Appendix 1: Detailed description of strapping techniques

### A1.1. Longitudinal strapping technique

- The shoulder area was prepared by wiping it with alcohol rub to enhance adhesiveness.
- The first layer of strapping was Fixomull® Stretch, followed by a layer of Leukotape P® which was applied with a cephalad tension.
- With the patient seated, the arm was positioned with a pillow beneath the elbow in an attempt to reduce any presence of subluxation.
- Three to four strips were applied, starting just below the deltoid insertion:
  ⁚ Anteriorly, over the glenohumeral joint to end on the spine of the scapula.
  ⁚ Posteriorly, over the glenohumeral joint to end on the mid-clavicle but before the suprasternal notch.
  ⁚ Laterally, over the glenohumeral joint to end just beyond the acromio-clavicular joint. This strip was omitted if the patient’s shoulder and upper arm were undersized.
  ⁚ A final strip was applied over the distal part of the three strips to secure them.
  The research assistant wrote on the tape ‘Do not wet; do not remove’ (Griffin & Bernhardt 2006).

### A1.2. Circumferential strapping technique

- The shoulder area was prepared by wiping it with alcohol rub to enhance adhesiveness.
- Fixomull® Stretch was used as the strapping material.
- With the patient seated, the arm was positioned with a pillow beneath the elbow in an attempt to reduce any presence of subluxation.
- Taping commenced along the length of the lateral half of the clavicle.
- The tape was then applied diagonally across the deltoid muscle, with a slight stretch applied in the same direction of the posterior fibres of deltoid. The stretch was not over-exerted as this could have caused vascular compression.
- The tape then travelled under the axilla, over padding material that was positioned on the inner surface of the upper arm for protection and comfort. The padding material did not extend the full way around the arm, as this was not necessary.
- The tape ended on the first quarter of the spine of the scapular.
- A second strip of tape was applied in the same way, but 2 cm inferiorly.
- The beginning and end of the taping were secured with a third strip of tape that ran over the shoulder.
- The research assistant wrote on the tape ‘Do not wet; do not remove’ (Ancliffe 1992; Griffin & Bernhardt 2006).
